# HDAC1 is involved in the destabilization of the HSF2 protein under nonstress and stress conditions

**DOI:** 10.1016/j.cstres.2025.100079

**Published:** 2025-05-01

**Authors:** Kevin Daupin, Véronique Dubreuil, Johanna K. Ahlskog, Annalisa Verrico, Lea Sistonen, Valérie Mezger, Aurélie de Thonel

**Affiliations:** 1 Université de Paris, CNRS, Epigenetics and Cell Fate, Paris, France; 2 ED 562 BioSPC, Université Paris Cité, Paris, France; 3 Faculty of Science and Engineering, Cell Biology, Åbo Akademi University, Turku, Finland; 4 Turku Bioscience Centre, University of Turku and Åbo Akademi University, Turku, Finland

**Keywords:** Heat shock factors, Histone/lysine deacetylases, Heat shock transcription factor 2 ubiquitin-proteasome degradation, Stress, Neurodevelopment, Human cerebral organoids

## Abstract

Heat shock transcription factors 1 and 2 (HSF1 and HSF2) are the major regulators of the cellular response to stressors, notably to heat shock and to oxidative stress. HSF1 and HSF2 are also important contributors in devastating human pathologies like cancer, neurodegenerative disorders, and neurodevelopmental disorders. Under physiological conditions, nuclear HSF2 is detected in only a few cell types in human adult healthy tissues. In contrast, HSF2 protein levels are elevated at some embryonic stages, but greatly vary among cell types and fluctuate during the cell cycle in diverse cell lines. HSF2 is a short-lived protein whose rapid turnover is controlled by the components of the ubiquitin-proteasome degradation pathway, and the stabilization of HSF2 constitutes an important step that regulates its DNA-binding activity and mediates its roles in nonstress, physiological processes. The control of HSF2 abundancy is therefore critical for its regulatory roles in stress responses as well as under physiological conditions. In this regard, the fetal brain cortex is a singular context where HSF2 is strikingly abundant, exhibits constitutive DNA-binding activity and, by controlling a specific repertoire of target genes that play important roles at multiple steps of neurodevelopment. Recently, we showed that the lysine-acetyl-transferases CBP and EP300 stabilize the HSF2 protein under both unstressed and stressed conditions and that the integrity of the CBP/EP300-HSF2 pathway is important for neurodevelopment. Here, we identify the lysine-deacetylase histone-deacetylase 1 (HDAC1) as a novel HSF2-interacting protein partner and regulator, in an unbiased manner, and show that HSF2 and HDAC1 localize in the same cells in the developing mouse cortex and human cerebral organoids. We also demonstrate that HDAC1, through its catalytic activity, destabilizes the HSF2 protein, through HSF2 poly-ubiquitination and proteasomal degradation, under both normal and stress conditions.

## Highlights

• Heat shock transcription factor (HSF2) is a short-lived protein degraded by the ubiquitin-proteasome pathway and stabilized by CBP/EP300-mediated acetylation.
• The control of HSF2 abundancy is critical for its roles under unstressed and stress conditions.
• Histone-deacetylase 1 as a protein partner of HSF2.
• Histone-deacetylase 1 promotes poly-ubiquitination and proteasomal HSF2 degradation under physiological and stress conditions.

## Graphical abstract

**Figure:**
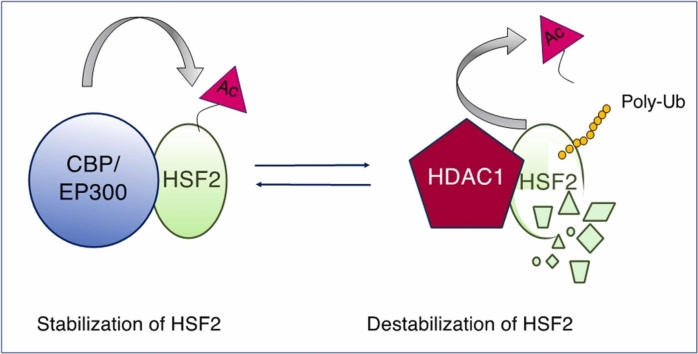

## Introduction

Rapid and significant changes in gene transcription represent a pivotal aspect of the cellular response to heat shock, that is, the heat shock response (HSR) and to its protective role of cell homeostasis. This protective effect operates through the activation of genes that encode molecular chaperones, including heat shock proteins (HSPs), in particular. Quick and sharp increase in the transcription of *HSP* genes, followed by their downregulation, is essential to restoration of proteostasis (protein homeostasis) and cell survival to stress. In a general point of view, rapid modulations of gene transcription rely on post-translational modifications of transcription factors, that ensure quick transduction of initial physiological or stress signals. In this regard, the acetylation of HSF1, the master regulator of the HSR^1^, ^2^ (Joutsen and Sistonen *et al.*, 2019^3^) modulates its DNA-binding activity and stability, which contributes to the exquisite regulation of HSP gene transcription^4^, ^5^, ^6^, ^7^ Among various post-translational modifications, HSF1 acetylation levels are the result of exquisite and complex balance between lysine-acetyl-transferase (KAT) and lysine-deacetylase (KDAC) activities. Historically, these enzymes were first identified through their ability to acetylate or deacetylate lysine residues in nucleosome histones and were thus called histone acetyl-transferases (HATs) and histone-deacetylases (HDACs), respectively. However, they also control the acetylation levels of transcription factors, such as HSF1, and, more broadly of a large repertoire of thousands of cellular proteins. Among KATs, CBP and EP300 form the unique KAT3 family, each of them (KAT3A and B, respectively) showing extensive sequence homology and displaying specific and overlapping functions in a number of cellular pathways, such as the regulation of enhancer-dependent, cell-type-specific, and stress-dependent gene transcription.^8^, ^9^, ^10^, ^11^, ^12^ EP300 participates in orchestrating the chronology of induction and attenuation of the HSR, by controlling the levels of HSF1 acetylation.^5^

HSF2 is a short-lived protein whose rapid turnover is controlled by the components of the ubiquitin-proteasome degradation pathway.^13^, ^14^, ^15^, ^16^, ^17^, ^18^ Accordingly, HSF2 abundancy is critical for its regulatory roles in the cell cycle and development under physiological conditions, as well as in stress responses^1^, ^14^, ^19^; HSF2 was first reported as a modulator of the HSR driven by HSF1. Classical acute heat shock provokes HSF2 rapid degradation and HSF2 only transiently modulates the activation of heat shock genes encoding the HSPs, whose stress-induced expression is predominantly driven by HSF1.^1^, ^20^ HSF2 protein levels fluctuate during the cell cycle, which further indicates that stabilization of HSF2 provides a critical control step in fine tuning the HSR.^21^, ^22^, ^23^ More generally, upon acute stress, HSF2 cooperates with HSF1, and their physical and/or functional interactions come in diverse flavors, in different cellular and physiological contexts. Although both factors recognize the same canonical heat shock element on DNA,^23^, ^24^ their cooperation leads to binding and regulation of distinct repertoires of genes and enhancers in a stress-type dependent manner, as was evident in the comparison of acute heat shock and oxidative stress.^24^ Importantly, HSF2 is critical in the response to stressors that are relevant for chronic or pathological situations, such as fever-like temperatures at 39-41 °C^25^ and prolonged proteasome inhibition.^14^, ^17^, ^26^ Furthermore, HSF2 is not degraded by chronic or acute exposure to alcohol and is the driver of this stress response and necessary for HSF1 activation in the developing brain, which was demonstrated in models of fetal alcohol syndrome.^27^, ^28^ HSF2 also plays important roles in cancer.^29^, ^30^, ^31^ HSF1 and HSF2 work in close physical and functional interaction across diverse types of cancer cell lines, HSF2 being a crucial partner of HSF1 in driving a specific transcriptional program that promotes malignancy and its anabolic demands.^31^ The control of HSF2 protein levels is therefore critical in a number of pathological situations.

Under physiological conditions, nuclear HSF2 is detected in only a few cell types in human adult healthy tissues.^32^ HSF2 protein levels greatly vary in diverse cell types or embryonic stages, and the stabilization of HSF2 constitutes an additional regulatory step for its DNA-binding activity and mediates its roles in non-stress, physiological processes.^33^, ^18^ In this regard, the fetal brain cortex is a singular context where HSF2 is strikingly abundant, exhibits constitutive DNA-binding activity and, by controlling a specific repertoire of target genes, plays important roles at multiple steps of neurodevelopment.^19^, ^27^, ^34^, ^35^, ^36^, ^37^ Altogether, the remarkable diversity in HSF2 abundance among cellular, tissue, developmental, and stress contexts, emphasizes the importance to decipher the molecular mechanisms triggering the proteasomal degradation of HSF2 or favoring its stability. We recently showed that CBP/EP300 acetylates HSF2 on three key lysine residues, which stabilizes this short-lived protein.^35^ We demonstrated that this molecular process is biological relevant for the integrity of neurodevelopment: HSF2 is acetylated in mouse fetal cortices and human cerebral organoids (hCOs), which are 3D neural models that recapitulate the early steps of neurodevelopment. Mutations of CBP or EP300 carried by patients with the neurodevelopmental disorder Rubinstein-Taybi syndrome (RSTS)^38^ lead to HSF2 degradation by the proteasome. We found that, as a consequence, HSF2 degradation was associated to abnormal chaperone equipment under normal (unstressed) conditions and neurodevelopmental phenotypes.^35^ We unraveled a CBP/EP300-HSF2-N-Cadherin cascade: the proteasome-dependent reduction of HSF2 levels in RSTS neural models leads to decrease N-Cadherin levels and disorganization of the neurogenic niche, signed by cell-cell adhesion defects.^35^ The HSF2 acetylation and stabilization by CBP/EP300 has thus important functional consequences. In agreement with our findings, a mutation in the *HSF2* gene, leading to HSF2 haploinsufficiency, was recently associated with a neurodevelopmental disorder called Angelman Syndrome-like,^39^ reinforcing the relevance of interrogating the mechanisms that control the dynamics of its stability and degradation in neurodevelopment and other pathophysiological situations. However, the control mechanisms underlying HSF2 deacetylation are not known.

Here, by characterizing the HSF2 protein interactome, using an unbiased TAP-TAG approach, coupled with mass spectrometry and cellular and biochemical analyses, we identify HDAC1 as a novel HSF2-interacting protein partner and regulator. Our results uncover that HDAC1 destabilizes the HSF2 protein, through HSF2 poly-ubiquitination and proteasomal degradation, under both normal and stress conditions.

## Results

### Unbiased identification of HDAC1 as a protein interacting with HSF2

**Fig. 1:**
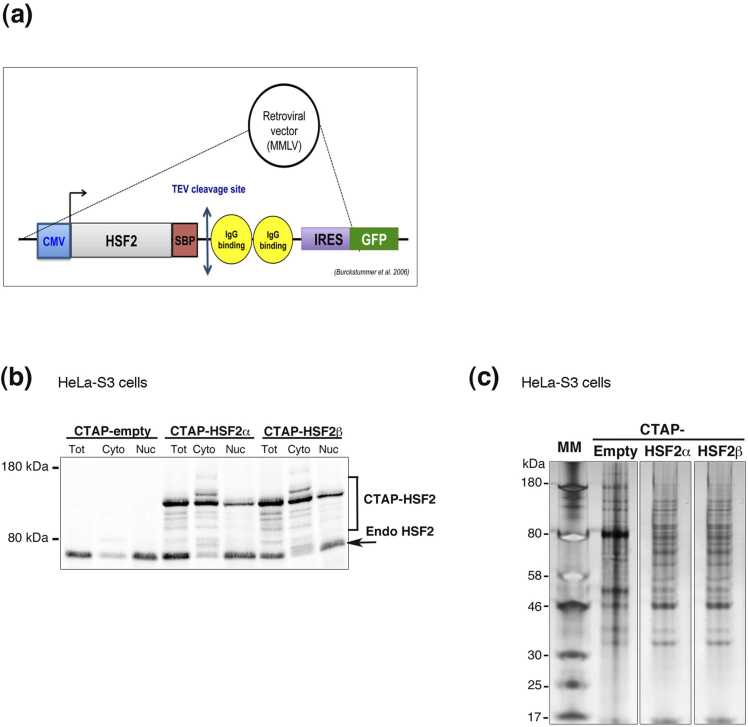
Strategy for the unbiased identification of HSF2 protein partners by TAP-TAG/MS analysis in HeLa-S3 cells. (related to Table 1). (a) Schematic representation of the construct generated for the identification of HSF2 nuclear partners by a dual affinity TAP-TAG (Tandem Affinity Purification Tag) approach in HeLa-S3 cells. HeLa-S3 cells were transfected with either control vector pCeMM CTAP (SG; that encodes streptavidin-binding peptide (SBP), TEV (Tobacco Etch Virus) cleavage sites, two IgG binding units of protein G, and the IRES-EGFP (Internal Ribosome Entry Site - Enhanced Green Fluorescent Protein only)^40^ or with PCEMM-CTAP-HSF2 (α or β), and the nuclear extracts were used for a dual affinity purification (GS-TAP) using the G-protein domains (in yellow) and streptavidin-binding peptide (SBP in red), sequentially. (b) HSF2 Western blot analysis of whole cell (Tot), cytosolic (Cyto), and nuclear (Nuc) extracts from the HeLa-S3-CTAP-empty, HeLa-S3-CTAP-HSF2α and HeLa-S3-CTAP-HSF2β cell populations. The exogeneous recombinant HSF2 has lower electrophoretic mobility. (c) SDS-PAGE silver staining of 5% of final double-TAP-TAG eluates from HeLa-S3-CTAP-empty, HeLa-S3-CTAP-HSF2α, and HeLa-S3-CTAP-HSF2β nuclear extracts. HSF, heat shock transcription factor; MM, molecular markers in kilodaltons (kDa); Internal Ribosome Entry Site-Enhanced Green Fluorescent Protein; SDS-PAGE; Sodium Dodecyl Sulfate - Polyacrylamide Gel Electrophoresis.

To identify HSF2-binding protein partners, we performed an unbiased screen, using a double-affinity TAP-TAG approach.^40^ For this purpose, we generated a HeLa-S3 cell line expressing double-tagged HSF2 (Figure 1(a)) or transfected with the empty vector as a negative control. The developing brain expresses two HSF2 splicing isoforms differing by the presence of the exon 11 HSF2α and HSF2β^33^, ^34^, ^41^, ^42^; each of which potentially sharing partners with the other and also having specific ones. Thus, we introduced either *Hsf2*α or *Hsf2*β cDNA in HeLa-S3 cells. By FACS and Western blot analyses, we selected among the GFP-positive Hela-S3 cell populations those that expressed similar amounts of exogenous recombinant HSF2 proteins in the nucleus, compared to the endogenous HSF2 levels, in order to minimize non-physiological effects that could result from massive nuclear HSF2 levels, due to overexpression, and potentially perturb the HSF2 interactome (Figure 1(b)). As expected, the recombinant protein was found both in the nuclear and cytoplasmic compartments at significant levels. We focused our identification of the HSF2 partners localized in the nucleus and therefore, potentially influencing the role of HSF2 as a transcription factor. The double-tagged HSF2 protein from nuclear extracts was submitted to two sequential purification steps, using affinity for IgG and Streptavidin, followed by mass spectrometry (MS) analysis (Figure 1(c)). Among the enriched HSF2 protein partners, we identified nucleoporin Nup62, a member of the nucleopore complex (Table 1), an already known HSF2 partner.^43^ We also identified HSF2 in this screen, indicating that HSF2 was able to interact with itself, which is coherent with HSF2 existing as homodimers or homotrimers (Table 1).^44^ The identification of both Nup62 and HSF2 validated the quality of our TAP-TAG/MS assay. Interestingly, we also identified HDAC1 as a novel HSF2-interacting protein (Table 1). Moreover, our data suggest that HDAC1 is able to interact with both HSF2α and β isoforms,since one HDAC1 peptide was common to the MS analyses involving these two isoforms (Table 1). Notably, we did not identify any other lysine deacetylases by this approach.

**Table 1:** Identification of HDAC1 as an HSF2 Protein Partner by TAP-TAG and Mass Spectrometry in Hela-S3 Cells.

**HSF2-alpha TAP-TAG + MS**											
**scanf**	**Rank**	**charge**	**Ions**	**dCn**	**dCn2**	**XCorr**	**Reference**	**Redu**	**Peptide**	**UniProt**	**Name**
3408	1	2	13/20	0.2446	0.2446	2.2287	UPSP:HSF2_HUMAN	2	K.QSSNVPAFLSK.L	Q03933	**Heat Shock Factor HSF2**
3744	1	2	15/22	0.1694	0.1694	2.4124	UPSP:HSF2_HUMAN	2	R.DGPVEFQHPYFK.Q		
3955	1	2	18/22	0.3347	0.3347	3.6184	UPSP:HDAC1_HUMAN	7	K.YGEYFPGTGDLR.D	Q13547	**lysine-deacetylase HDAC1**
6479	1	2	19/26	0.1372	0.1372	3.8995	UPSP:NUP62_HUMAN	3	K.ELEDLLSPLEELVK.E	P37198	**Nuclear pore glycoprotein p62**

**HSF2-beta TAP-TAG + MS**											

**scanf**	**Rank**	**charge**	**Ions**	**dCn**	**dCn2**	**XCorr**	**Reference**	**Redu**	**Peptide**	**UniProt**	**Name**

2950	1	2	15/20	0.4034	0.4034	2.8991	UPSP:HSF2_HUMAN	2	K.QSSNVPAFLSK.L	Q03933	**Heat Shock Factor HSF2**
3440	1	2	16/22	0.3078	0.3078	3.2013	UPSP:HDAC1_HUMAN	7	K.YGEYFPGTGDLR.D	Q13547	**lysine-deacetylase HDAC1**

**Identification of HDAC1 and HDAC2 as HSF2 Protein Partners by co-Immunoprecipitation and Mass Spectrometry in E17 fetal cortices**											

**IP mouse cortex E17 + MS**											

**scanf**	**Rank**	**charge**	**Ions**	**dCn**	**dCn2**	**XCorr**	**Reference**	**Redu**	**Peptide**	**UniProt**	**Name**

4186	1	3	37/72	0.4438	0.4438	6.1421	Hdac2_IPI:IPI00137668.1	1	K.LHISPSNM*TNQNTPEYM*EK.I	P70288	**lysine-deacetylase HDAC2**
6240	1	3	36/88	0.4273	0.4273	4.6291	Hdac2_IPI:IPI00137668.1	1	K.VM*EM*YQPSAVVLQCGADSLSGDR.L		
3131	1	2	10/14	0.1904	0.1904	1.7987	Hdac2_IPI:IPI00137668.1	1	K.YHSDEYIK.F		
5773	1	2	18/22	0.3659	0.3659	3.2165	Hdac1_IPI:IPI00114232.1	5	K.YGEYFPGTGDLR.D	O09106	**lysine-deacetylase HDAC1**
6366	1	3	27/48	0.2375	0.2375	4.1463	Hdac1_IPI:IPI00114232.1	5	R.M*THNLLLNYGLYR.K		
6501	1	2	19/22	0.3261	0.3261	3.3955	Top2a_IPI:IPI00122223.1	1	K.ELILFSNSDN	Q01320	**DNA Topoisomerase 2a**
6732	1	2	20/24	0.3432	0.3432	3.5990	Top2a_IPI:IPI00122223.1	1	K.IFDEILVNAADNK.Q		
4817	1	2	09/12	0.0565	0.0565	1.6952	Top2a_IPI:IPI00122223.1	1	K.IVGLQYK.K		
5668	1	2	16/24	0.2414	0.2414	3.3872	Top2a_IPI:IPI00122223.1	1	K.QIM*ENAEINNIIK.I		
6055	1	2	09/12	0.2269	0.2269	1.9211	Top2a_IPI:IPI00122223.1	11	K.YGVFPLR.G		
6578	1	2	12/18	0.2094	0.2094	1.8525	Top2a_IPI:IPI00122223.1	1	R.EVTFVPGLYK.I		
7268	1	2	17/20	0.3158	0.3158	3.0910	Tln1_IPI:IPI00465786.3	2	K.GLAGAVSELLR.S	P26039	**Talin-1**
8300	1	3	28/72	0.4769	0.4769	3.6665	Ywhae_IPI:IPI00118384.1		K.AAFDDAIAELDTLSEESYK.D	P62259	**Ywhae 14-3-3 protein epsilon**
4691	1	2	12/20	0.2866	0.2866	1.9925	Ywhae_IPI:IPI00118384.1		K.EAAENSLVAYK.A		
5089	1	2	19/24	0.4433	0.4433	3.2126	Ywhae_IPI:IPI00118384.1	2	K.VAGM*DVELTVEER.N		

Identification of HDAC1 as an HSF2 Protein Partner by TAP-TAG and Mass Spectrometry (MS) in Hela-S3 Cells and by co-Immunoprecipitation and Mass Spectrometry in E17 fetal cortices. (Upper Panels) After sequential immunoprecipitation of nuclear extracts of HeLa-S3 expressing CTAP-HSF2α and CTAP-HSF2b, using two tags (G-protein and Streptavidin-binding peptide; see Figure 6A), eluates were analyzed by MS. (Lower panel) After immunoprecipitation of HSF2 from extracts of fetal cortices at gestational day 17 (E17), eluates were analyzed by MS). The number of unique peptides from each identified protein and their UniProt Knowledgebase (UniProtKB) codes are indicated

TAP-TAG, Tandem Affinity Purification tag.

### HDAC1, HDAC2, and HSF2 profiles along mouse cortical development and hCO differentiation

**Fig. 2:**
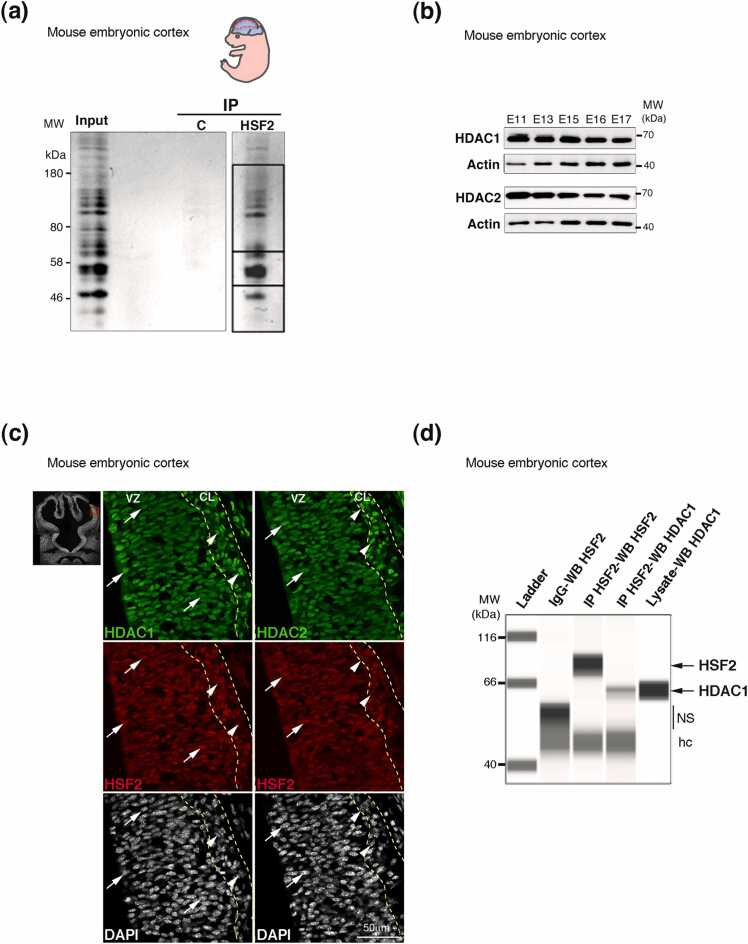
HDAC1 and HSF2 are coexpressed in cells of the mouse cortex and hCOs. (a) Unbiased identification of HSF2 protein partners by coimmunoprecipitation and MS in the mouse cortex. SDS-PAGE (Sodium Dodecyl Sulfate - Polyacrylamide Gel Electrophoresis) colloidal blue staining of HSF2 immunoprecipitated proteins from lysates of cortices at embryonic day 17.5 (E17) prior MS analysis. The rectangles delimit the gel parts that were cut and subjected to MS analysis. (b) HDAC1 and 2 proteins are expressed at all stages of mouse cortical development. Representative immunoblots of protein extracts from E11 telencephalon (Tel) and E13 to E17 mouse cortex, showing the expression profiles of HDAC1 and HDAC2 (n = 2 independent experiments). Actin serves as a loading control. (c) Coexpression of HSF2 and HDAC1/HDAC2 in the cells of the E12.5 mouse cortex. The left panel points to the cortical region magnified in the right panels**.** Representative confocal immunofluorescence of mouse developing cortex at embryonic day 12.5 (E12.5), showing that HSF2 (red) and HDAC1/HDAC2 (green) are expressed in the DAPI-stained nuclei of neural progenitor cells, characterized by their radial organization, and of neurons, identified by their tangential orientation. Left, apical side; right, basal side. VZ, ventricular (proliferative) zone; CL, cortical (neuronal) layer, forming the CP. Each panel is 70 µm wide (n = 2 independent experiments). The dashed-yellow line indicates the limits between the VZ and the CL. (d) HDAC1 is coimmunoprecipitated with HSF2 in the E12.5 mouse cortex. Representative experiment of Simple Western™ protein separation and immunodetection of HSF2 and HDAC1 using ProteinSimple’s Jess™ (see Materials and Methods). NS, nonspecific signal; hc, IgG heavy chains. MW, molecular weights. n = 2 technical replicates.

HSF2 is present and acetylated in the mouse prenatal cortex and in hCOs, where we showed that it influences neurodevelopment (see introduction). In addition to the TAP-TAG/MS approach in the non-neural cell line HeLa S3, we thus performed another unbiased experiment to identify HSF2 protein partners by immunoprecipitation of the endogenous HSF2 protein in whole-tissue extracts of mouse cortices at embryonic day 17, (E17; Figure 2(a)), and subsequent MS analysis. We detected proteins that were previously identified in HSF2 coimmunoprecipitation experiments in the mouse testis and/or prostate cancer PC-3 cells,^45^ reinforcing the physiological relevance of our HSF2 coimmunoprecipitation experiment: among them, DNA topoisomerase TOP2A, YWHAE (14-3-3 protein epsilon) and Talin-1 (Table 1), which was validated as a HSF2 partner by orthogonal methods^45^ and is involved in the development of neurites.^46^ Notably, we also found that not only HDAC1, but also HDAC2, coimmunoprecipitated with HSF2 (Table 1), whereas we did not identify any other lysine deacetylases.

We therefore examined HDAC1, HDAC2 and HSF2 expression profiles in these two tissues. HDAC1 and HDAC2 were expressed all along mouse cortical development, as shown in Western blot experiments (Figure 2(b)). To examine the cellular localization of HDAC1, HDAC2, and HSF2 in neural structures, we focused on mouse fetal cortices at embryonic day 12.5 (E12.5), a stage at which dividing neuroprogenitor cells (NPCs) represent the major cell populations and at which their progeny, the young postmitotic neurons, starts to be produced. Immunostaining of the mouse cortex at E12.5 revealed that HDAC1 and HDAC2 were present in virtually all nuclei of NPCs, which are radially organized cells, located in the proliferative layer, called the ventricular zone (VZ) (Figure 2(c), arrows). Both HDAC1 and HDAC2 were also detected in the majority of neurons, whose nuclei are often tangentially orientated and located within the cortical plate (CP; Figure 2(c), arrowheads). Note that HDAC2 is enriched in CP cells, as indicated by its increased staining intensity (Figure 2(c), arrowheads). At this stage, HSF2 was similarly localized in the nuclei of both NPCs and neurons (Figure 2(c)). Moreover, we further validated the interaction of HSF2 and HDAC1 by showing that HDAC1 coimmunoprecipitated with HSF2 in lysates of the E12 mouse cortex (Figure 2(d)).

**Fig. 3:**
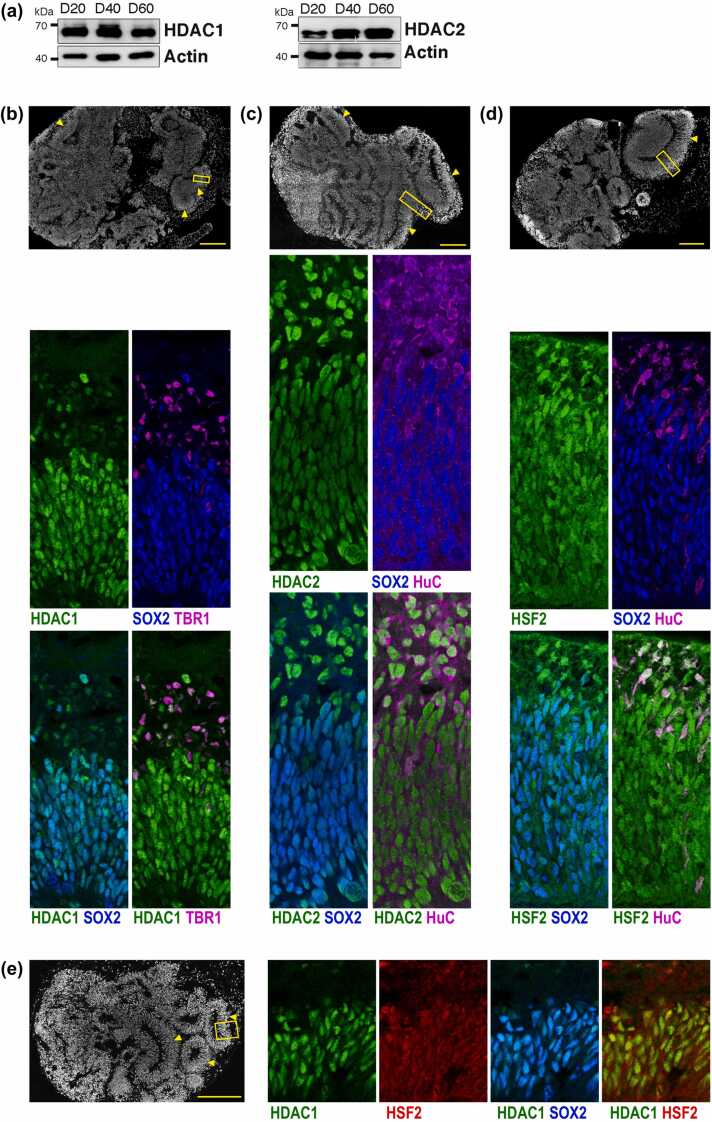
HDAC1 and HSF2, and HDAC2 and HSF2 are coexpressed in cells of hCOs. (a) Representative immunoblots of protein extracts of D20, D40, and D60 hCOs, showing the expression profiles of HDAC1 and HDAC2. Actin serves as a loading control. n = 2 experiments. (b-d) (Upper panels) Representative reconstruction of confocal microscopy images of D25 hCOs from the healthy donor IMR90-4 iPSC line (see Materials and methods), showing that hCOs present with multiple cortical-like regions (“loops”; yellow arrowheads). Scale bar, 200 µm. The yellow rectangle points to the region magnified in the middle and lower panels. (Middle and lower panels) Confocal localization of HDAC1 (green, b), HDAC2 (green, c), and HSF2 (green, d) in proliferative NPCs, labeled with SOX2 (blue) and young neurons labeled with TBR1 or HuC/D (purple). HDAC1 is present in NPCs and almost absent from young neurons, while HDAC2 is weakly expressed in NPCs and more in young neurons. HSF2 is present in both progenitors and neurons, and enriched in the latter. (e) Colocalization of HDAC1 (green) and HSF2 (red) in proliferative NPCs (SOX2, purple). Note that HSF2 is not only nuclear but also cytoplasmic, while HDAC1 is strictly nuclear. Although HSF2 and HDAC2 are present in most of the nuclei of NPCs and neurons, suggesting that they colocalize in at least a certain number of nuclei, we were not able to illustrate it by colabeling. This limitation is due to a lack of primary antibodies, compatible with the use of secondary antibodies from different species. Each panel in (b-e) is 70 µm wide.

We then explored the expression profiles of HDAC1, HDAC2 and HSF2 in the to-date best proxy of human early neurodevelopment, hCOs, which recapitulate cell diversity and organization of the fetal brain (Lancaster *et al.*, 2013).^47^, ^48^ ^49^ We found that HDAC1 and HDAC2 were expressed from day in vitro 20 (D20) to D60 of hCO differentiation (Figure 3(a)), as well as HSF2.^35^ Consistently with seminal studies,^49^ hCOs at D25 contained cortex-like structures (“loops,” organized around a lumen; Figure 3(b-d), upper panels and Figure 3(e), left panel; arrowheads). We showed that, as expected, these cortical-like loops are organized like the neocortex, that is into a VZ-like layer of NPCs, (expressing the NPC marker SOX2), surrounded by a CP-like layer of neurons (expressing the neuron markers TBR1 and HuC/D); Figure 3(b-d), middle and lower panels; Lancaster et al., 2013). HDAC1 was detected in virtually all nuclei of SOX2+ NPCs (Figure 3(b) and (e), middle and lower panels) and in TBR1+ neurons at lower levels than in NPCs (Figure 3(b), middle and lower panels). This pattern contrasted with the E12.5 mouse cortex, where HDAC1 was seen enriched in neurons (Figure 2(c)). HDAC2 staining was also observed in the nuclei of SOX2+ NPCs and HuC/D+ neurons, but its staining was more intense in neurons than in NPCs, as observed in the mouse cortex (Figure 3(c), middle and lower panels, see Discussion). HSF2 was also localized in the nuclei of NPCs (Figure 3(d), middle and lower panels), as previously reported in older D60 hCOs.^35^ HSF2 and HDAC1 were thus codetected in the vast majority of nuclei of NPCs in hCOs (Figure 3(e)). Akin to HDAC2, HSF2 was expressed and slightly enriched in most HuC/D+ neurons (Figure 3(d)). Altogether, HSF2 and HDAC1 are present in the nuclei of NPCs in the mouse cortex as well as in hCOs, which is congruent with our identification of HDAC1 as an HSF2 protein partner in our proteomics analyses of Hela S3 cells and mouse cortex. This strengthens the biological relevance of this interaction. In line with our proteomics data in the mouse cortex, HDAC2 and HSF2 also exhibit colocalization in the mouse cortex and in hCOs, especially in postmitotic neurons. Interestingly, we also detected HDAC3 in the E11-E17 mouse cortex, as well as in hCOs (Supplementary Figure 1), and HDAC3 expression was reported in HeLa S3 cells.^50^ However, HDAC3 did not coimmunoprecipitate with HSF2, neither in our TAP-TAG/MS approach in HeLa S3 cells, nor in our HSF2 IP/MS or HSF2 IP/immunodetection experiments in the mouse cortex.

### Validation of HDAC1 as a protein partner of HSF2

**Fig. 4:**
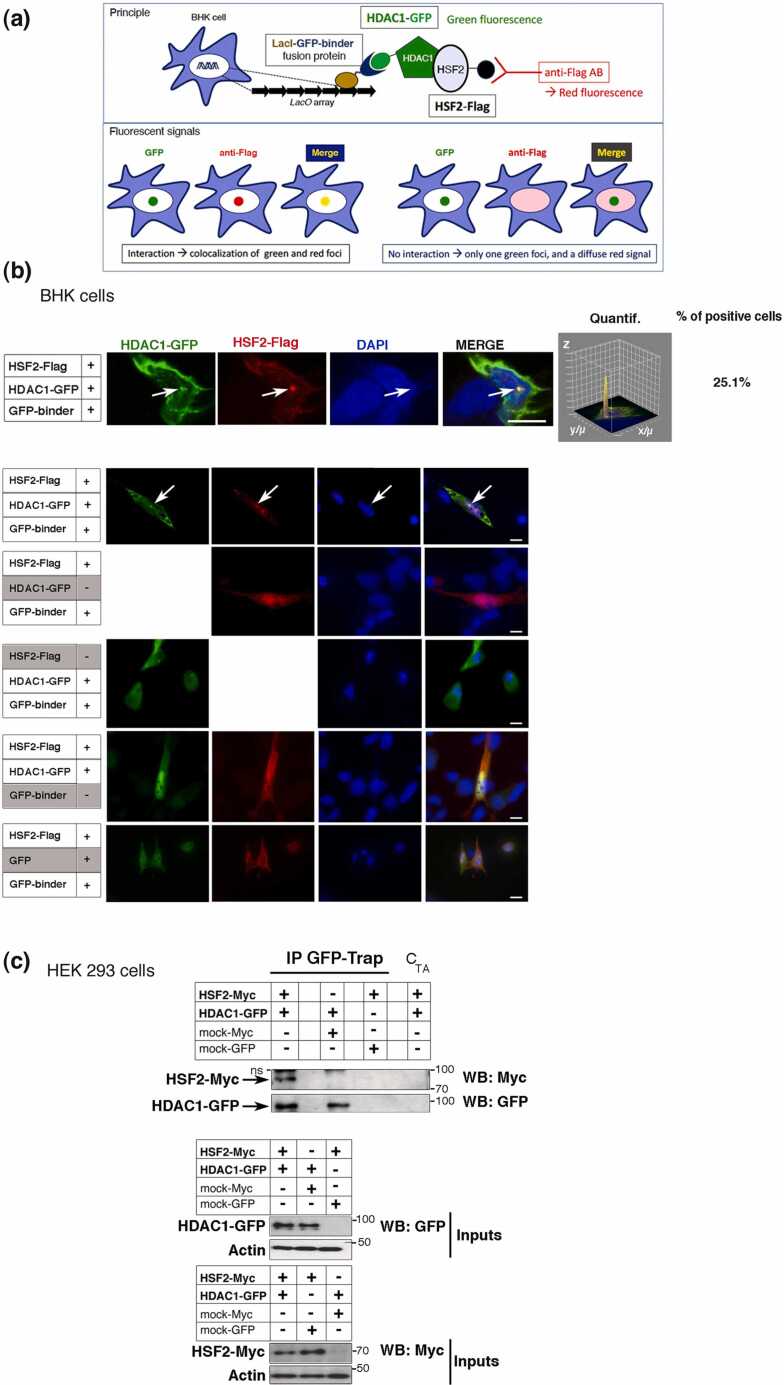
HDAC1 interacts with HSF2 in normal conditions. (a) Schematic representation of the principle of the fluorescent-3-hybrid (F3H) assay. Genomic integration of a *Lac0p* array allows the focal recruitment in the nucleus of a LacI fused to the GFP binder, which in turn recruits the GFP-tagged probe (HDAC1-GFP) and its potential interactants (HSF2-Flag). (b) Visualization of the interaction between ectopically expressed HDAC1-GFP and HSF2-Flag in F3H assays. (Upper panels) Confocal sections of BHK cells carrying a stably integrated Lac-operator array that were triple transfected with LacI fused to the GFP binder, HDAC1-GFP, and HSF2-Flag constructs, or double transfected with a combination of two of these constructs as indicated. Chromatin was counterstained using DAPI. Scale bar, 5 µm. (Lower panels) F3H control experiments for the visualization of the interaction between HSF2-Flag and exogenous HDAC1-GFP or GFP. n = 3 independent experiments. Scale bar, 10 µm. (c) The ectopically expressed exogenous HDAC1 interacts with HSF2. (Upper panel) GFP-Trap coimmunoprecipitation of HDAC1-GFP and HSF2-Myc in transfected HEK 293 cell extracts. (Middle and lower panels) Immunoblot showing total HDAC1 (WB GFP) or HSF2 levels (WB Myc) in inputs, respectively. n = 3 independent experiments. Actin was used as a loading control. ns, nonspecific band.

We then examined the interaction between HSF2 and HDAC1 *in cellulo*. For this, we used the fluorescent three-hybrid assay (F3H) established in BHK cells.^51^ BHK cells carry genomic integration of a *Lac0p* array that allows the focal recruitment in the nucleus of a LacI fused to a Green Fluorescent Protein (GFP) binder, which, in turn, recruits the GFP-tagged probe (here, HDAC1-GFP) and any potential interactant—here, Flag-HSF2 (schematic illustration, Figure 4(a)). In this assay, HDAC1-GFP was recruited by a GFP binder to the *Lac0p* array locus (green spot) to which Flag-HSF2 was found corecruited, in 25% of the cases (red spot; Figure 4(b), upper panel). All the negative controls failed to exhibit such colocalization, as expected (Figure 4(a), lower panels). HDAC1 and HSF2 colocalization occurred in a limited percentage of BHK cells (Figure 4(c)), which might be either due to the highly transient nature of their interactions or to the artificial nature of the cell system. Therefore, we further explored the HSF2-HDAC1 interaction in another cell line, using a biochemical approach. Using coimmunoprecipitation in GFP-Trap assays upon coexpression of HDAC tagged with GFP and HSF2-Myc fusion proteins, we confirmed that HSF2 and HDAC1 were able to interact in the mammalian HEK 293 cell line (Figure 4(c)).

### HDAC1 promotes HSF2 deacetylation

**Fig. 5:**
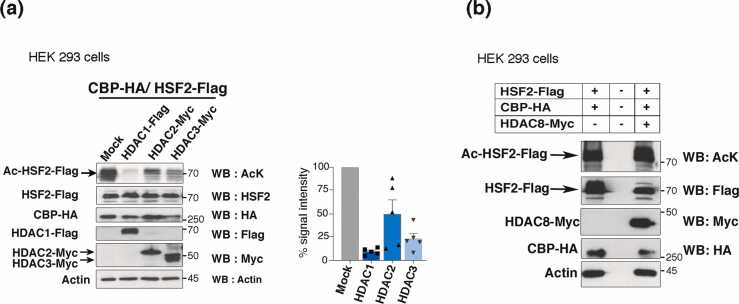
Overexpression of HDAC1 markedly reduces the acetylation of HSF2 by CBP. (a) (Left panels) HEK 293 cells were transfected with the following constructs: CBP-HA and HSF2-Flag, and HDAC1-Flag, HDAC2-Myc, or HDAC3-Myc and the acetylation status of the HSF2-Flag protein was checked by using an anti-AcK antibody. (Right panel) Quantification of the signal intensity, normalized to the “Mock”-transfected samples. n = 5 independent experiments. Error bars, mean ± SEM. Actin was used as a loading control. (b) same as in (a) but HEK 293 cells were transfected with CBP-HA and HSF2-Flag, and HDAC8-Myc. n = 3 independent experiments. NB: The HSF2 signal is often detected as several bands of different molecular weights in diverse cell lines or tissues and is variable in biological or technical replicates; in particular, they are possibly due to post-translational modifications.

We investigated the impact of HDAC1 and other class I HDACs on the acetylation of HSF2 in HEK 293 cells coexpressing CBP-HA and HSF2-Flag with the various HDAC proteins (Figure 5(a)). Western blot analysis revealed robust acetylation of HSF2, in the presence of CBP, as previously demonstrated.^35^ This allowed us to detect it without immunoprecipitation. However, we verified that the signal detected with a pan acetyl-lysine antibody specifically corresponded to the acetylation of the HSF2-Flag and not of another protein, as shown in samples cotransfected with both CBP-HA and HSF2-Flag. Indeed, in the range of migration of the full-length HSF2-Flag protein, acetylation was neither detectable in the absence of exogenously expressed CBP-HA nor when CBP-HA was expressed alone (Figure 5(a); Supplementary Figure 2). On this basis, we observed that HDAC1 overexpression resulted in reduction in HSF2 acetylation levels, which is compatible with a role of HDAC1 in HSF2 deacetylation. Notably, HDAC2 and HDAC3 were also able to deacetylate HSF2 (Figure 5(a)), whereas HDAC8 had hardly detectable effects (Figure 5(a) and (b)).

### HDAC1 promotes HSF2 degradation upon heat shock

**Fig. 6:**
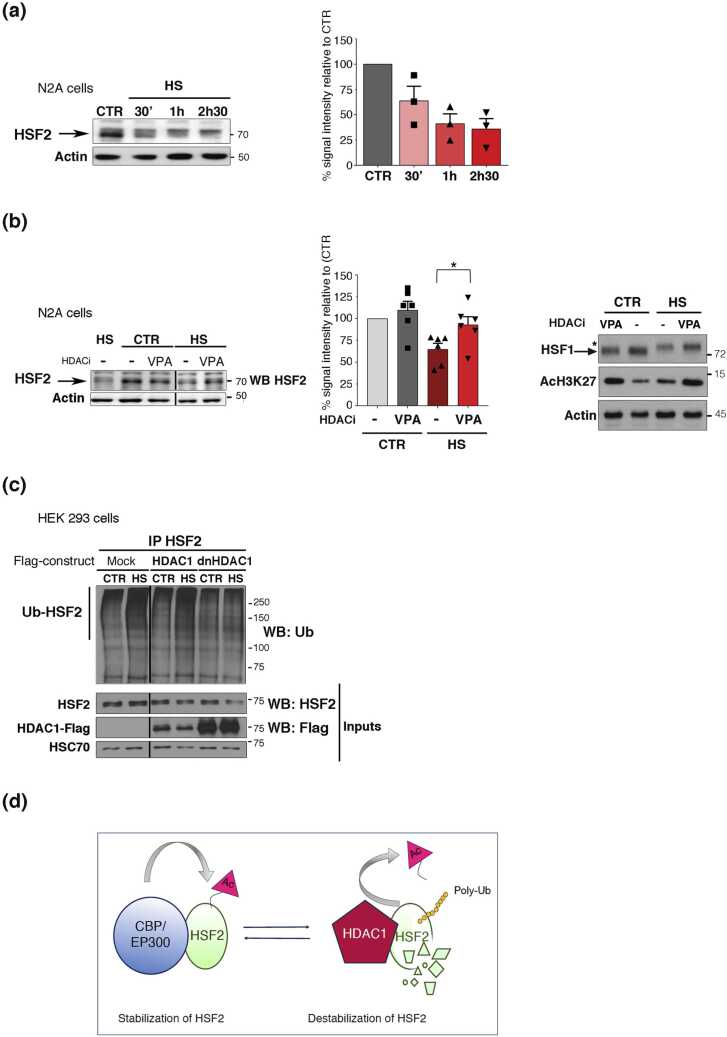
HDAC1 promotes HSF2 degradation upon heat shock. (a) Endogenous HSF2 levels are decreased upon HS in N2A cells. Representative immunoblot and quantification of n = 3 independent experiments. Error bars, mean ± SEM. (b) Class I HDAC inhibitor VPA prevents the decrease in endogenous HSF2 protein levels induced by HS. N2A cells were pretreated or not with 1 mM VPA for 3 h and subjected to HS 42 °C for 2h30. (Left panel) Representative immunoblot. (Middle panel) Quantification on the signal intensity normalized to actin levels (n = 4 independent experiments). Error bars, mean ± SEM, *P < 0.05. (Right panel) Assessment experiment for the efficiency of the HDAC inhibitor, VPA, in N2A cells. Representative immunoblot for H3K27 acetylation (AcH3K27). n = 3 independent experiments. (c) Expression of dominant-negative HDAC1 prevents the accumulation of poly-ubiquinated HSF2 upon HS. HEK 293 cells were cotransfected with HSF2-Myc, EP300-HA, and Flag-HDAC1 or dnHDAC1, and subjected or not to HS 42 °C (30 min). Representative immunoblot of poly-ubiquinated HSF2 levels after Myc-HSF2 immunoprecipitation. n = 3 independent experiments. (d) Scheme of the model of HSF2 dynamic balance between acetylation by CBP/EP300 and deacetylation by HDAC1 and its consequences in terms of stabilization or degradation.

We previously showed that heat shock provokes the degradation of HSF2 and that mimicking the acetylation of the three major acetylated lysine residues in the HSF2 protein counteracts its heat shock-induced degradation.^35^, ^13^ We therefore investigated the impact of the inhibition class I HDACs on the levels of endogenous HSF2 protein in the neural progenitor cell line N2A. We first showed that heat shock was also able to induce HSF2 decay in N2A cells, although it occurred at a slower rate than in HeLa or HEK 293 cells (Figure 6(a)).^13^ Treatment with 1 mM of the class I HDAC inhibitor valproic acid (VPA) dampened the decline in HSF2 protein levels in N2A cells exposed to HS (Figure 6(b)). This indicates that class I HDAC activity participates to the degradation of HSF2 upon heat shock, probably through HSF2 direct deacetylation. We thus verified first that heat shock increased HSF2 poly-ubiquitination in HEK 293 cells, as previously reported^13^ (Figure 6(c), mock transfection). Next, to investigate whether HDAC1 could favor HSF2 poly-ubiquitination, we examined the impact on HSF2 ubiquitination of overexpressing a dominant-negative form of HDAC1 mutated in its deacetylase catalytic site. We showed that, indeed, the increase in HSF2 poly-ubiquitination induced by heat shock was mitigated in HEK 293 cells transfected with dominant-negative HDAC1 (Figure 6(c), compare lanes “HDAC1” and “dnHDAC1”).

As a whole, our results support a role of HDAC1 (and possibly other class I HDACs) in the destabilization of HSF2 under normal and stress conditions, through HSF2 poly-ubiquitination and proteasomal degradation, likely following the deacetylation of HSF2.

## Discussion

HSFs are involved in a wide spectrum of pathophysiological situations, in which one member of the HSF family works either alone or in combination with another. Moreover, the deregulation of HSF activities has been incriminated in the onset and progression of severe diseases including neurodevelopmental, neurodegenerative disorders and cancer, which represent a worldwide human, societal and economic burden.^39^, ^35^, ^19^, ^1^, ^22^ However, the mechanisms by which the expression levels of HSFs are regulated in a context-dependent manner have remained poorly studied.

Here, we identify, in an unbiased manner, interactions between HDAC1 and HSF2 in HeLa S3 cells as well as mouse cortices and, between HDAC2 and HSF2, in the mouse cortex only. The expression patterns of HDAC1, and HDAC2 are consistent with potential interactions with HSF2 in human cortical organoids. We found that overexpression of either of these two HDACs leads to HSF2 deacetylation. We confirm that HDAC1 interacts with HSF2, both in coimmunoprecipitation followed by immunodetection in the mouse cortex, as well as, in *in cellulo* F3H assays (but we failed to capture HSF2-HDAC2 interaction in these assays). Then, focusing on HDAC1, which we identify as a robust HSF2 partner in the diverse cells and human and mouse neural tissue models and as able to deacetylate HSF2, we demonstrate that HDAC1 catalytic activity is needed for the degradation of HSF2 in a ubiquitin-proteasomal dependent manner. This mechanism may provide an explanation for the rapid degradation of HSF2 by APC/C in response to heat shock,^13^ since, in particular, we find that the expression of a dominant-negative mutant of HDAC1 prevents the induction of HSF2 poly-ubiquitination induced by heat shock.

Importantly, in our TAP-TAG/Mass Spectrometry (MS) approach, we have selected cells that express exogenous HSF2 at levels as similar as possible as those of the endogenous nuclear HSF2 protein. However, the exogenous HSF2 cytoplasmic levels turned out to be higher than the endogenous HSF2 ones, in those cells, which could have influenced the results. First, the fact that HDAC1 is nuclear should minimize such perturbations if any. Second, this is why we challenged these results by diverse approaches including IP/MS analysis and F3H assays. More physiologically, we also identified HDAC1 (and HDAC2) as an HSF2 protein partner in the prenatal mouse cortex upon by HSF2 IP/MS analyses. In that case, not only it was performed on the endogenous HSF2 pool, but also in a context where HSF2 plays important roles and is known to be acetylated reviewed in.^19^, ^35^

HDAC1 and HDAC2 are important epigenetic enzymes that control key steps of neurodevelopment: double deletion of either HDAC1 or HDAC2 leads to complex defects in neurogenesis, the positioning of neurons and neuronal differentiation.^52^, ^53^, ^54^, ^55^, ^56^, ^57^ The fine tuning of HSF2 protein levels is also necessary for these neurodevelopmental processes.^19^ Thus, HDAC1 (and to a lesser extent, HDAC2) may also have important potential pathophysiological consequences, not only through its direct role on the chromatin landscape, but also by tightly controlling the levels of HSF2 in a context-dependent manner, especially during neurodevelopment. In line with this, we found coexpression of HSF2 and HDAC1 in the nuclei of NPCs in the mouse developing cortex, as well as in hCOs, in which the HSF2 protein is known to exert important roles reviewed in.^19^, ^35^ To our knowledge, our study provides the first data on the pattern of expression of HDAC1, HDAC2 proteins, (as well as HDAC3), in hCOs at stages that mimic early prenatal development (D20-D60), thus completing a study that mainly focuses on human cortical organoids at later stages equivalent to the postnatal period^58^ and a study that reported the presence of HDAC1, 2, and 3 transcripts in DIV 35 hCOs*.*^59^ Notably, in our hands, HDAC1 and HDAC2 staining at E12.5 exhibited the same pattern in NPCs and in neurons of the mouse cortex, as described by MacDonald and Roskam^55^ and Tang *et al.*^57^ HDAC2 staining was more intense in neurons than in NPCs in the mouse cortex as well as in hCOs. We observed differences in HDAC1 expression between the two neural models and at the chosen stages of analysis. Indeed, at later stages, at E13.5 and E16.5, when the CP start to become thicker, HDAC1 expression in neurons is weaker compared to NPCs, comparable to the staining observed in hCOs.^55^^,^^57^ This suggest that the role of HDAC1 and HDAC2 in regulating proteasomal degradation of HSF2 might differ in a cell-type-dependent manner and or stage-dependent manner during neurodevelopment.

In terms of pathology, the impact of HDAC1 on HSF2 in the RSTS context is unclear. Of note, we did not observe any differences in HDAC1 levels by comparing primary fibroblasts from RSTS patients to those from healthy donors,^35^ but clearly observed a decrease in the acetylation levels of histone residue H3K27, which suggests that there is no compensation for the lack of CBP/EP300 activity by reduction of HDAC1 levels or activities in RSTS cells. In addition, we showed that VPA, an inhibitor of class I HDACs, was unable to restore HSF2 levels in RSTS cells, whereas it increases HSF2 levels in healthy donor counterparts.^35^ One interpretation is that CBP/EP300 is the major KAT acetylating HSF2 in these cells. In addition, in this CBP/EP300-deficient cells, HSF2 cannot be acetylated, and thereby does not constitute a proper substrate for HDAC1 and any other class I HDACs, which eventually renders HDAC inhibition ineffective on HSF2.

The other class I HDACs, HDAC2 and HDAC3 might also be involved in the deacetylation and degradation of HSF2. Interestingly, HDAC3 is also expressed throughout mouse neocortex prenatal neurodevelopment and the perinatal period at neurogenic stages (E12.5 to P0), and is involved in the control of neurogenesis (Nodwood *et al.*, 2014).^60^, ^61^ Our data (Supplementary Figure 1) are thus congruent with these reports. Notably, treatment by VPA that inhibits HDAC1, 2, and 3, perturbs the number of NPCs and neuronal differentiation with different impacts depending on the stage considered and time-window of exposure, and the model (human cortical organoids-on-a-chip and in hCOs^62^ (Kelaa *et al.*, 2022). Similar results were obtained using specific inhibitors of HDAC1 and HDAC3 and HDAC2 and HDAC3, respectively (Kelava *et al.*, 2022). In addition, VPA rescues neurodevelopment in human brain organoids modeling a lysosomal neurologic disorder.^63^ Altogether, these findings suggest that HDAC3 plays a role in brain organoids and our data further characterize the expression profile of HDAC3 in early hCOs. However, as for its role in HSF2 deacetylation, we could not identify HDAC3 as a HSF2 protein partner in our two unbiased strategies, although it is expressed in HeLa S3 cells and the mouse, nor could we coimmunoprecipitate it with HSF2 in the mouse prenatal cortex. Further studies in other cell systems would be needed to clarify this point. Finally, we do not exclude the possibility that class III HDACs, in particular SIRT1, which deacetylates HSF1,^6^ could be involved in HSF2 deacetylation. However, neither SIRT1 nor other members of the class III NAD-dependent HDACs were detected in our study of the HSF2 interactomes in HeLa-S3 cells or in the mouse cortex. In addition, in our hands, treatment with NAM, a Sirtuin inhibitor, failed to induce reproducible elevation of HSF2 protein levels nor increase in HSF2 acetylation by CBP/EP300 (J.A. and L.S., unpublished results). Future studies are warranted to determine whether other enzymes (HATs/KATs or HDACs) regulate HSF2 acetylation, deacetylation and thereby its stability or activity, and modify other lysine residues that we found acetylated in our MS analysis,^35^ as it is the case for HSF1.^4^, ^5^, ^6^, ^64^

HDAC1 is known to deacetylate other transcription factors and modulate their stability or activity (e.g., p53, E2F, and YY1).^65^ Besides the effects of HDAC1 on HSF2 stability, the association of HSF2 with HDAC1 might also have a role in gene regulation by acting on and within the chromatin context. Indeed, HSF2 has a versatile — active or negative — impact on transcription, by acting as an activator of repressor, depending on the target gene and cellular state and environment considered cells lines, cancer cell lines, stress conditions, differentiation stages etc.^66^, ^24^, ^31^

## Conclusion

Overall, it is likely that HSF2 undergoes rapid cycles of acetylation and deacetylation, because CBP/EP300, HSF2, and HDAC1 are found in the nuclei of the same progenitor cells in the mouse cortex as well as in hCOs, and the dynamics of this process, and likely rapid degradation and stabilization of HSF2 might *per se* be important for neurodevelopment, as it is the case during the cell cycle.^21^, ^22^, ^23^

In addition, our findings are of importance in other contexts that potentially includes other cell types, tissues, but also stress conditions and pathologies. First, they provide a better understanding of the rapid degradation of HSF2 that occurs during heat shock: we previously showed that acetylation prevents HSF2 to be degraded not only under unstressed conditions, but also upon heat shock^35^ and this study fills a gap in this mechanism by showing that HDAC1 is a major lysine-deacetylase that, through HSF2 deacetylation, mediates HSF2 proteasomal degradation upon heat shock. Second, the property of HSF2 to be dynamically degraded or stabilized may represent an advantage for tumor cells to adjust to abrupt proteotoxic conditions that they undergo, and our findings might provide a better understanding of this dynamics in the future.^67^, ^31^

## Materials and methods

More detailed information and requests for resources and reagents should be directed to and will be fulfilled by the cocorresponding authors: Valérie MEZGER (valerie.mezger@u-paris.fr), Lea SISTONEN (lea.sistonen@abo.fi), and Aurélie de Thonel (aurelie.dethonel@u-paris.fr),

## Cell lines and culture

Cell culture, transfections and treatments: murine Neuro2A (N2A, neuroblastoma, DSMZ # ACC 148), Hamster BHK (kindly provided by Dr Leonhardt H and cultured as described,^51^ human HEK 293 T (ATCC®, CRL-11268™), and HeLa-S3 cells (kindly provided by Dr Slimane Ait-Si-Ali, and cultured as described^68^) were grown in DMEM (Lonza Group Ltd.) supplemented with 4,5 g/L glucose and 10% fetal bovine serum (FBS, Life technology) in humidified atmosphere with 5% CO2 at 37 °C. IMR-90 iPSCs were grown in mTesR plus (Stem Cell Technologies) on plates coated with Matrigel (Corning). The conditions of treatment of cells by heat shock or valproic acid treatment are indicated in Figure 5.

## Production of hCOs

iPSCs were differentiated into hCOs, using the STEM diff™ cerebral organoid kit, according to the manufacturer guidelines (Stem Cell Technologies), as described in de Thonel *et al.*^35^

## Mouse model

Specific pathogen-free C57BL/N female mice were purchased from Janvier (Lyon, France) and maintained in sterile housing in accordance with the guidelines of the *Ministère de l′Enseignement Supérieur et de la Recherche* (Paris, France). Rodent laboratory food and water were provided ad libitum. Experiments were performed in accordance with French and European guidelines for the care and use of laboratory animals.

## Reagents and cell treatments

Proteasome inhibitor MG132 was used for 6 h at a final concentration of 20 μM. HDACs inhibitor VPA (Interchim, AYJ060) was used at 1 mM for 3 h, in N2A cells. For all chemicals, Dimethyl sulfoxide was used as vehicle (control).

Heat shock was performed in water bath at 42 °C for the indicated times.

## Antibodies

**Figure:**
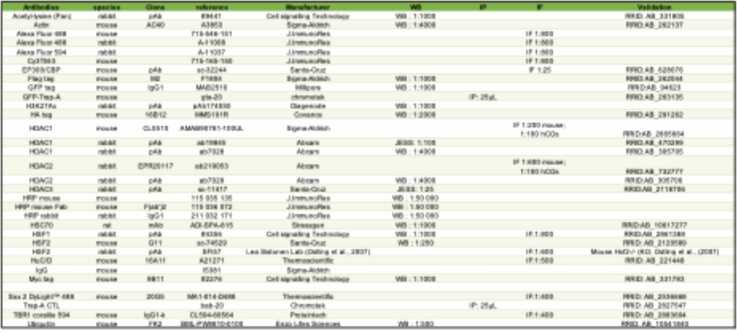

## Plasmids and constructs

For Tandem Affinity Purification (TAP), *Hsf2α* and *Hsf2β* cDNA, derived from E16.5 mouse brain mRNAs, were inserted in vector PCEMM-CTAP (Euroscarf P30536) with CMV-driven expression of insert and GFP (Green Fluorescent Protein) used as an indirect reporter (IRES (Internal Ribosome Entry Site-Enhanced)). Retrovirus production and cell transduction was performed as described.^69^ GFP-positive clones were then isolated by clonal dilution, selected by FACS and amplified.

**Figure:**
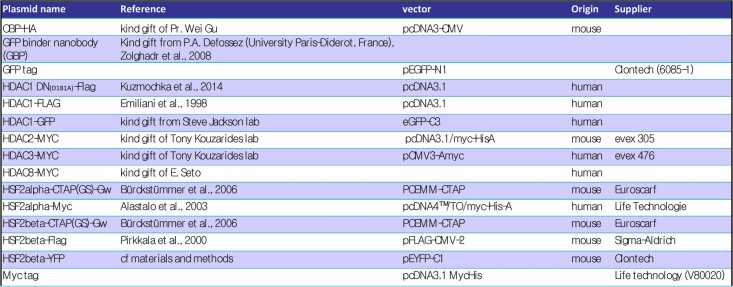

## Transfection of HEK cells

HEK 293 cells were transfected using XtremGENE HP Reagent (Sigma-Aldrich) according to the manufacturer's instructions, with a combination of Myc- or Flag-tagged hHSF2 and the specified HDAC construct, CBP-HA, EP300-HA, or a mock vector.

## Fluorescence three-hybrid assay

Fluorescence three-hybrid assay (F3H) was performed according to Herce *et al*. and de Thonel *et al*.^51^, ^35^ BHK cells were transfected with constructs expressing YFP-HSF2, CBP-HA, or EP300-HA, and GBP-LacI, using different combinations (ratio 1:1.5:2) at 70-80% confluency using reverse transfection by Lipofectamine 2000 (Thermo Fisher Scientific), as indicated. Medium was changed after 4 h for all transfections. After 24 h, the cells were fixed in 4% PFA on coverslip and stained with mouse anti-HA (Covance) or rabbit anti-CBP antibody (Santa-Cruz), followed by a staining with mouse or rabbit fluorescent secondary antibody (Jackson Immunoresearch), respectively. Confocal microscopy images were taken on a confocal microscope Leica TCS SP5 (*IMAGOSEINE* Imaging Platform in Institut Jacques Monod) and images were analyzed using Fiji software (ImageJ2 v2;3;0/1.53k).

## TAP-TAG and mass spectrometry analysis

Hela-S3, stably expressing GS-HSF2α, GS-HSF2β or the empty PCEMM-CTAP (SG) vector, were grown in floating cultures (spinners) to obtain large quantities of materials and 10 g per cell line was collected. Note that, among the GFP-positive cell populations, we selected by combining FACS and Western blot analyses, those exhibiting levels of the recombinant HSF2 proteins similar to that on the endogenous HSF2 protein. Nuclear extracts were prepared from 3 × 10^9^ of these selected cells, as described before except that no DNase treatment was applied.^70^ The quality of the nuclear fraction was assessed by their enrichment in histones as observed after staining of the electrophoresis gels by Coomassie blue. Total nuclear extracts were incubated with IgG-agarose beads overnight, transferred to Poly-prep columns (Bio-Rad), washed four times in wash buffer (Tris 50 mM, NP40 0.1%, 150 mM), and then once in TE buffer (Tris 1M (pH8), EDTA 10mM). Elution was made by incubating the beads twice with Tobacco Etch Virus enzyme (Invitrogen) for 45 min. Eluates were collected in Tobacco Etch Virus buffer and then incubated with Dynabeads My-one streptavidin for 1 h, washed once, and eluted using D-biotin 5 mM. Proteins were then concentrated using TCA/acetone precipitation and resuspended in Laemmli buffer. Protein identification by MS (LC/MS/MS) was carried out at the TAPLIN Biological Mass Spectrometry Facility (Harvard Medical School, Boston, MA, USA).

## Immunoprecipitation and MS analysis of the endogenous HSF2 protein from E17 mouse cortices

We also analyzed by MS the partners of the endogenous protein HSF2 from E17 fetal cortices. Brain cortices were mechanically lysed into 4.5 volumes of the following buffer: 10 mM HEPES (pH 7.9), 400 mM NaCl, 5%, glycerol, 100 mM EGTA) and two cycles of freezing and thawing in liquid nitrogen, and centrifuged at 20,000 g for 30 min. The HSF2 protein was immunoprecipitated in supernatants, by using a monoclonal antibody (clone 3E2, Abcam) or PBS as a negative control, and protein G agarose (Roche). Proteins that coimmunoprecipitated with HSF2 were analyzed on Sodium Dodecyl Sulfate - Polyacrylamide Gel Electrophoresis gel bands (staining with colloidal blue) and sent to TAPLIN Biological Mass Spectrometry Facility (Harvard Medical School, Boston, MA, USA) for MS (LC/MS/MS) analysis.

## Immunoprecipitation, Western blotting, and Jess analyses

For Western blots of cells and hCOs, protein extracts were prepared using a modified Laemmli buffer (5% sodium dodecyl sulfate, 10% glycerol, 32.9 mM Tris-HCl pH 6.8) supplemented with protease inhibitors (Sigma-Aldrich). For Western blots, brain tissues were prepared with a lysis buffer (Hepes 10 mM pH 7.9; NaCl 0.4 M, EGTA 0.1 M; glycerol 5%, dithiothreitol [DTT] 1 mM, PMSF 1 mM, protease inhibitor (Sigma-Aldrich), phosphatase inhibitor [Roche]). Then, 20 μg of proteins from lysates were subjected to migration on 8-12% acrylamide gels and transferred on to polyvinylidene difluoride membranes (GE Healthcare Europe GmbH) in borate buffer (50 mM Tris-HCl and 50 mM borate) for 1 h 45 at constant voltage (48 V). The membranes were incubated with primary antibodies overnight at 4 °C, then washed in Tris-buffered saline-Tween 0.1% and incubated for 1 h with horseradish peroxidase (HRP)-coupled secondary antibody (Jackson Immunoresearch). The signal was revealed using a chemiluminescent reagent (Pierce® ECL Plus Western Blotting Substrate, Thermo Scientific) and was detected using hyperfilm (HyperfilmTM ECL, Amersham Biosciences) and a film processor (Konica Minolta). Poly-ubiquitinated HSF2 was detected as described in Ahlskog *et al.*^13^ Immunoprecipitation experiments were performed as described in.^35^ Alternatively, protein samples were analyzed using a fully automated and capillary-based immunoassay using ProteinSimple’s Jess™ Simple Western™ instrument, according to the manufacturer guidelines (Bio-Techne). The denatured samples were diluted in the sample buffer (Protein Simple, #042-195) to a concentration of 0.25 μg/μL. Three microliters were subjected to migration *via* capillary gel electrophoresis, and proteins of interest were detected using primary antibodies and manufacturer-provided specific secondary antibodies. Total protein loaded was assessed using a total protein staining reagent (DM-TP01, Bio-Techne), which was utilized for normalization. The independent capillaries were then analyzed with dedicated Protein Simple software (Compass).

## Immunoprecipitation of exogenous proteins, using GFP/Myc-Trap

Cells were lysed in Lysis buffer (50 mM Hepes pH 8, 100 mM NaCl, 5 mM EDTA, Triton X-100 0.5%, Glycerol 10%, VPA (1 mM), DTT 1 mM, PMSF 1 mM, proteases inhibitors, phosphatase inhibitors [Roche]) and then, HDAC1 was immunoprecipitated using anti-GFP-trap antibody, or as a control Trap®-A control (ChromoTek). GFP-Trap®-A (ChromoTek) enables purification of any protein of interest fused to GFP. Immunoprecipitated proteins were run on an 8% SDS-polyacrylamide gel, followed by an immunodetection of HSF2 by protein using anti-Myc antibody.

## Immunofluorescence in hCOs

hCOs were fixed as described in Lancaster and Knoblich.^71^ hCOs were then embedded in PolyFreeze Tissue Freezing Medium (Sigma-Aldrich; SH0026) for cryoprotection and stored at −80 °C. hCOs were cryosectioned into 12 µm thick slices and disposed on Superfrost™ Adhesion Plus slides, then stored at −20 °C before immunofluorescence.

Antigen retrieval was performed using Universal HIER antigen retrieval reagent (Abcam; ab208572). Slices were let to cool down and washed in PBS, then saturated for at least 1 h for with 3% fetal bovine serum (FBS) in 0.1% PBS-Triton X100 and incubated with primary antibody overnight at 4 °C. After washing in PBS-Triton X 100, slices were incubated with corresponding secondary antibody (Jackson ImmunoResearch) for up to 1 h at room temperature. Slices were then incubated with DAPI (100 ng/ml in PBS) for 15 min before being washed in PBS and then mounted in Fluoromount ™ (Sigma-Aldrich; F4680). Primary antibodies: HSF2 (H57,^66^; 1:800); HDAC1 (Sigma-Aldrich, AMAB90781 dilution, 1:200 for mouse tissue; dilution, 1:100 for hCOs); HDAC2 (Abcam, ab219053; dilution, 1:600 for mouse tissue and 1:100 in hCOs), HuC/D (Invitrogen, A-21271—1:500); SOX2 (Thermo Fischer Scientific, MA1-014-D488—1:400); TBR1 (Proteintech, CL594-66564—1:400) (see also Table 1 in the “Antibody” section).

Images presented in Figures 2 and 3 were acquired by confocal microscopy using a LSM900 from Zeiss (EPI^2^ *Epifluorescence for Epigenetics* Platform at UMR7216) and processed on ZEN 3.5 (Blue edition) and/or Image J and/or Adobe Illustrator.

## Statistics

Data are displayed as means ± standard error of the mean (SEM). GraphPad Prism 8 (GraphPad Software, La Jolla, CA, USA) was used for statistical analyses. Statistical significance was assessed using the Mann-Whitney test for two groups. Statistical tests are two-sided. *P*-values below 0.05 are considered statistically significant.

## Ethics statement

Our research complies with all relevant ethical regulations for the boards/committees and institutions that approved the study protocols. The use and storage of iPSCs were approved by the "*Cellule de bioéthique, Direction générale de la recherche et de l′Innovation*" at the French *Ministère de l′Enseignement Supérieur et de la Recherche* (MESRI), which delivered the CODECOH agreement (DC-2021-4446). iPSCs IMR90-4 were commercial purchased from WiCell, USA; MTA 21-W0506 and derive from a healthy donor (female; fetal). The above-cited CODECOH agreement DC-2021-4446) by the "*Cellule de Bioéthique*” also approved the use of these commercial iPSCs from WiCell.

The use of a mouse model in this study has been approved by the Animal Experimentation Ethical Committee Buffon (CEEA-40) and recorded under the following reference by the *Ministère de l′Enseignement Supérieur, de la Recherche et de l′Innovation* (#2016040414515579).

## Funding and support

VM was funded by the CNRS (*Projet International de Coopération Scientifique* PICS 2013-2015) for her collaboration with LS and by the Short Researcher Mobility France Embassy/MESRI-Finnish Society of Sciences and Letters; the *Agence Nationale de la Recherche* (HSF-EPISAME, SAMENTA ANR-13-SAMA-0008-01), and *Fondation Jérôme Lejeune* (2014-2015). LS was funded by the Academy of Finland, Sigrid Jusélius Foundation, Magnus Ehrnrooth Foundation and Cancer Foundation Finland. KD by was supported by PhD Fellowships the French Ministry of *Enseignement Supérieur et de la Recherche* and *Fondation pour la Recherche Médicale* (FRM). JKA was supported by Magnus Ehrnrooth Foundation. The supporting bodies played no role in any aspect of study design, analysis, interpretation, or decision to publish this data.

## Author contributions

AdT, LS, VD, and VM designed the work. AdT, JA, KD, and VD performed experiments and data analyses. VM wrote the manuscript with inputs from AdT, KD, VD, JA, and LS. All the authors read and approved the final manuscript.

## CRediT authorship contribution statement

**Daupin Kevin:** Writing – review & editing, Formal analysis, Data curation. **Verrico Annalisa:** Investigation, Supervision. **Mezger Valerie:** Writing – review & editing, Writing – original draft, Validation, Supervision, Funding acquisition, Conceptualization. **de Thonel Aurélie:** Writing – review & editing, Validation, Formal analysis, Data curation, Conceptualization. **Sistonen Lea:** Writing – review & editing, Funding acquisition. **Ahlskog Johanna K:** Writing – review & editing, Formal analysis. **Dubreuil Véronique:** Writing – review & editing, Formal analysis, Data curation, Conceptualization.

## Declarations of interest

The authors declare the following financial interests/personal relationships which may be considered as potential competing interests. Valerie MEZGER reports financial support was provided by Jérôme Lejeune Foundation. Kevin DAUPIN reports financial support was provided by Foundation for Medical Research. If there are other authors, they declare that they have no known competing financial interests or personal relationships that could have appeared to influence the work reported in this paper.

## Data Availability

Data will be made available on request.

## Footnotes

Appendix A Supplementary data associated with this article can be found online at doi:10.1016/j.cstres.2025.100079.
