# Supplementary material for: HDAC1 is involved in the destabilization of the HSF2 protein under nonstress and stress conditions
Source: Cell Stress Chaperones. 2025 May 1;30(4):100079. doi: 10.1016/j.cstres.2025.100079 (PMC12150047; doi:10.1016/j.cstres.2025.100079)
Supplement: Supplementary file 1 — Supplementary material [file mmc1.docx]

| **Plasmid name** | **Reference** | **vector** | **Origin** | **Supplier** |
| --- | --- | --- | --- | --- |
| CBP-HA | kind gift of Pr. Wei Gu | pcDNA3-CMV | mouse |  |
| GFP binder nanobody (GBP) | Kind gift from P.A. Defossez (University Paris-Diderot, France), Zolghadr et al., 2008 |  |  |  |
| GFP tag |  | pEGFP-N1 |  | Clontech (6085-1) |
| HDAC1 DN(D181A)-Flag | Kuzmochka et al., 2014 | pcDNA3.1 | human |  |
| HDAC1-FLAG | Emiliani et al., 1998 | pcDNA3.1 | human |  |
| HDAC1-GFP | kind gift from Steve Jackson lab | eGFP-C3 | human |  |
| HDAC2-MYC | kind gift of Tony Kouzarides lab | pcDNA3.1/myc-HisA | mouse | evex 305 |
| HDAC3-MYC | kind gift of Tony Kouzarides lab | pCMV3-Amyc | human | evex 476 |
| HDAC8-MYC | kind gift of E. Seto |  | human |  |
| HSF2alpha-CTAP(GS)-Gw | Bürckstümmer et al., 2006 | PCEMM-CTAP | mouse | Euroscarf |
| HSF2alpha-Myc | Alastalo et al., 2003 | pcDNA4™/TO/myc-His-A | human | Life Technologie |
| HSF2beta-CTAP(GS)-Gw | Bürckstümmer et al., 2006 | PCEMM-CTAP | mouse | Euroscarf |
| HSF2beta-Flag | Pirkkala et al., 2000 | pFLAG-CMV-2 | mouse | Sigma-Aldrich |
| HSF2beta-YFP | cf materials and methods | pEYFP-C1 | mouse | Clontech |
| Myc tag |  | pcDNA3.1 MycHis |  | Life technology (V80020) |
