# Supplementary material for: HDAC1 is involved in the destabilization of the HSF2 protein under nonstress and stress conditions
Source: Cell Stress Chaperones. 2025 May 1;30(4):100079. doi: 10.1016/j.cstres.2025.100079 (PMC12150047; doi:10.1016/j.cstres.2025.100079)
Supplement: Supplementary file 3 — Supplementary material [file mmc3.docx]

**SUPPLEMENTAL DATA**

**Supplementary Figure S1.** (related to Figures 2 and 3)


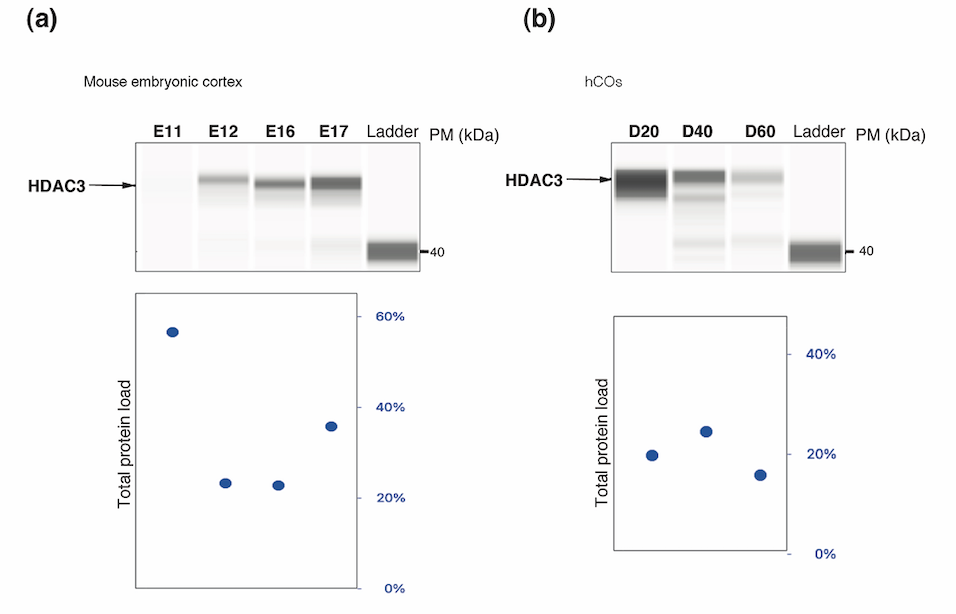


**HDAC3 is expressed throughout mouse cortical development and in early hCOs.**

(upper panels) Representative experiments of the detection of HDAC3 in lysates of mouse prenatal cortices at stages ranging from E11 to E17 stages (a) and D20 to D60 hCOs (b) using Simple Western™ protein separation and immunodetection using ProteinSimple’s Jess™ (see Materials and Methods). PM, molecular weights in kiloDaltons (kDa). (lower panels). Blue dots represent the relative amount of protein loaded in each capillary, expressed as a percentage of the detection signal and were used as loading controls.

**Supplementary Figure S2.** (related to Figure 5)


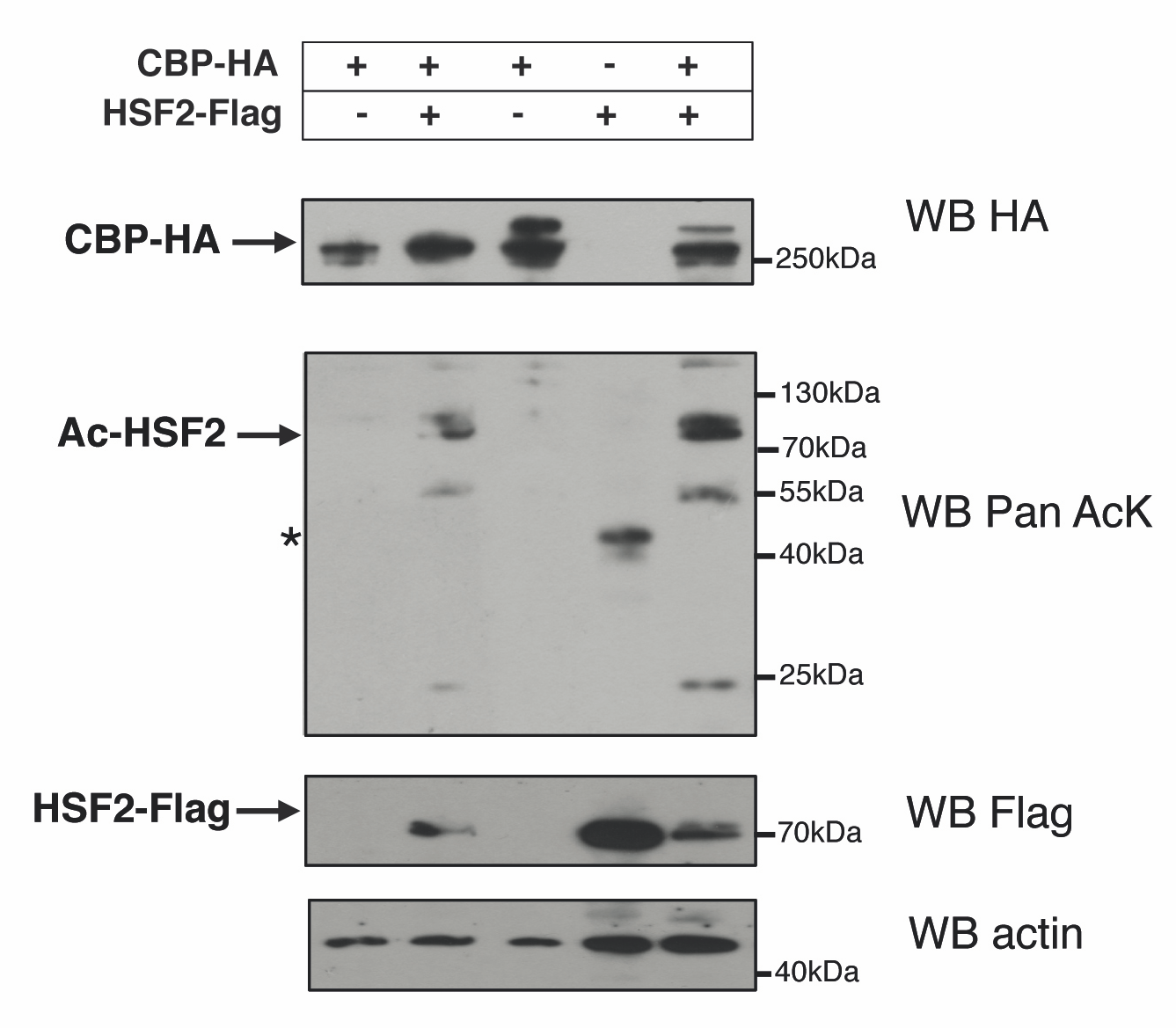


**LEGENDS OF SUPPLEMENTAL FIGURE S2**

**HSF2 acetylation is lost in the absence of CBP**

HEK 293 cells were transfected with the following constructs: CBP-HA and HSF2-Flag and the acetylation status of the HSF2-Flag protein was checked by using a Pan anti-AcK antibody. n = 3 independent experiments. The asterisk points to a 43-kDa band that was non-reproducibly detected in biological or technical replicates, and possibly corresponds to an acetylated degradation product of the full-length HSF2 or to a non-specific band. Actin was used as a loading control.
